# Gender perception as a predictor of women’s attitudes towards infertility: a cross-sectional study

**DOI:** 10.1186/s12905-026-04906-0

**Published:** 2026-09-21

**Authors:** Gülay Can, Çiler Yeyğel

**Affiliations:** 1 Çerkeş Community Health Center, Republic of Turkey Ministry of Health, Çankırı, Turkey; 2 Faculty of Health Science, Midwifery, İzmir Tınaztepe University, İzmir, Turkey

**Keywords:** Infertility, Gender perception, Attitudes, Women's health, Reproductive health

## Abstract

**Background:**

Infertility is not only a medical condition but also a reproductive health issue with significant psychological, social, and cultural consequences for women. Gender perception may influence women’s attitudes toward infertility; however, evidence regarding this relationship remains limited. This study aimed to examine the relationship between gender perception and attitudes toward infertility among women and to determine whether gender perception predicts attitudes toward infertility.

**Methods:**

This cross-sectional study included 346 women aged 18–60 years who were registered at the Çerkeş Family Health Center. Data were collected using the Introductory Information Form, the Infertility Attitude Scale, and the Gender Perception Scale. Descriptive statistics, independent samples t-test, one-way analysis of variance, Pearson correlation analysis, and multiple linear regression analysis were used to examine the association between gender perception and attitudes toward infertility while accounting for relevant sociodemographic and pregnancy-related characteristics.

**Results:**

The mean Infertility Attitude Scale score was 48.23 ± 7.55, and the mean Gender Perception Scale score was 97.45 ± 18.22. A moderate positive correlation was found between attitudes toward infertility and gender perception (*r* = 0.634, *p* < 0.001). Multiple linear regression showed that gender perception was positively associated with attitudes toward infertility (B = 0.262, β = 0.621, 95% CI: 0.218–0.306, *p* < 0.001), with the model explaining 46.8% of the variance (R² = 0.468; adjusted R² = 0.440). Employment status, marital status, and pregnancy history were also significantly associated with infertility attitudes (*p* < 0.05).

**Conclusions:**

Gender perception was positively associated with women’s attitudes toward infertility. These findings highlight the potential value of gender-sensitive approaches in reproductive health education and counselling.

**Supplementary Information:**

The online version contains supplementary material available at https://doi.org/10.1186/s12905-026-04906-0.

## Background

Infertility is a disease of the reproductive system defined as the failure to achieve pregnancy after 12 months or more of regular unprotected sexual intercourse [1]. Affecting millions of people worldwide, infertility is a major global public health problem that affects not only reproductive capacity but also psychological, social, and economic well-being and quality of life [1, 2]. The experience of infertility cannot be understood independently of the sociocultural context in which individuals live [3, 4]. In societies where motherhood is closely linked to women’s identity and social status, infertility may carry greater social and psychological consequences for women [3]. Therefore, infertility should be considered not only a disease of the reproductive system requiring medical management but also a complex women’s health issue with psychological, social, and cultural dimensions [1, 2].

Gender norms are among the key social determinants influencing individuals’ health behaviours and health-seeking practices [5]. Gender roles and beliefs about motherhood may also shape women’s psychological well-being and their attitudes towards reproductive health [6]. Likewise, women’s experiences of infertility are strongly influenced by socially constructed gender roles and cultural expectations surrounding motherhood [7]. Although infertility affects both partners, its psychosocial burden and social consequences continue to fall disproportionately on women in many societies [8].

These societal expectations may contribute to stigma, feelings of inadequacy, guilt, social isolation, and psychological distress among women experiencing infertility [9–11]. Recent evidence has shown that gender norms play a critical role in women’s access to sexual and reproductive health services, utilisation of these services, and reproductive health outcomes [12, 13]. Therefore, addressing infertility from a gender perspective is essential for a better understanding of women’s experiences of infertility and the gender-related inequalities that influence reproductive health [4].

Knowledge and awareness of infertility are important factors influencing individuals’ attitudes towards infertility, health-seeking behaviours, and engagement with infertility treatment [14]. Recent studies have also suggested that gender perception and attitudes towards gender roles are associated with attitudes towards infertility [15, 16]. Although this relationship has been examined in previous research, evidence from community-based samples of women remains limited, particularly regarding the extent to which the association persists when relevant sociodemographic and reproductive characteristics are considered simultaneously [15–17]. Examining these factors together may provide a more contextualised understanding of the relationship between gender perception and attitudes towards infertility among women in the community. Such evidence may also help clarify whether the observed association extends beyond differences related to sociodemographic and reproductive characteristics.

Accordingly, this study aimed to examine the association between women’s gender perception and attitudes towards infertility in a community-based sample of women, while accounting for relevant sociodemographic and pregnancy-related characteristics. The study also examined whether gender perception and attitudes towards infertility differed according to sociodemographic and obstetric characteristics.

## Methods

### Study design and ethical approval

This cross-sectional descriptive study was conducted between 26 April and 1 September 2023 at the Çerkeş Family Health Center in Çankırı, Türkiye. The study was conducted in five Family Medicine Units affiliated with the health center and included women attending the center for primary healthcare services as well as women recruited through community-based health screening activities conducted in rural areas. Ethical approval was obtained from the Çankırı Karatekin University Health Sciences Ethics Committee (Meeting No: 6, Decision No: 2023/28 − 03, dated 28 March 2023). The study was conducted in accordance with the principles of the Declaration of Helsinki, and written informed consent was obtained from all participants prior to data collection.

### Study population and inclusion/exclusion criteria

The study population consisted of approximately 3,500 women aged 18–60 years who were registered at the Çerkeş Family Health Center and resided in the district centre of Çerkeş, Saçak Town, 49 villages, and 47 neighbourhoods in Çankırı, Türkiye. The required sample size was calculated using the known population sample size formula with a 95% confidence level and a 5% margin of error. The calculation indicated that a minimum sample of 346 participants was required.

A non-probability convenience sampling method was used. Women who attended the five Family Medicine Units of the Çerkeş Family Health Center for primary healthcare services and women encountered during community-based health screening activities in rural areas were invited to participate if they met the eligibility criteria. Women who voluntarily agreed to participate were included in the study. Eligibility criteria included being aged 18–60 years, being able to read and understand Turkish, and voluntarily agreeing to participate. Women who did not meet the eligibility criteria or declined to participate were excluded. A total of 346 women who met the eligibility criteria and provided written informed consent were included in the study. All participants who completed the data collection forms were included in the statistical analyses.

### Data collection

Data were collected between 26 April and 1 September 2023 through face-to-face interviews conducted by the researchers. Prior to data collection, women who met the eligibility criteria were informed about the purpose and procedures of the study. Written informed consent was then obtained from all participants. Data were collected using the Introductory Information Form, the Infertility Attitude Scale, and the Gender Perception Scale. The Introductory Information Form was developed by the researchers for this study based on the relevant literature [7, 8, 14, 18] and comprised 13 questions covering sociodemographic, obstetric, and infertility-related characteristics (Supplementary File 1). Each interview lasted approximately 10–15 min.

#### Infertility Attitude Scale (ATIS)

The Infertility Attitude Scale was developed by Siyez et al. in Türkiye to assess attitudes toward infertility. The scale consists of 12 items rated on a five-point Likert scale. Higher total scores indicate more positive attitudes towards infertility. In the original scale development and validation study, exploratory factor analysis supported a one-factor structure comprising 12 items, while confirmatory factor analysis provided evidence of a good model fit. Item analysis and discriminant validity were also examined. Cronbach’s alpha coefficients were 0.83 and 0.85 across the two validation studies [19]. In the present study, Cronbach’s alpha was 0.778, indicating acceptable internal consistency in the current sample.

#### Gender Perception Scale (GPS)

The Gender Perception Scale was developed by Altınova and Duyan to assess individuals’ gender perceptions [20]. The scale consists of 25 items rated on a five-point Likert scale, including 10 positively worded and 15 negatively worded items. Total scores range from 25 to 125, with higher scores indicating a more positive gender perception. Negatively worded items were reverse scored before calculating the total score. The scale was developed and validated in the Turkish context. In the original validation study, construct validity was examined using principal components analysis, and the results supported a unidimensional structure comprising 25 items. The Cronbach’s alpha coefficient was 0.872 [20]. In the present study, Cronbach’s alpha was 0.910, indicating high internal consistency in the current sample.

### Statistical analysis

All statistical analyses were performed using IBM SPSS Statistics for Windows, version 22.0 (IBM Corp., Armonk, NY, USA). Descriptive statistics were presented as mean ± standard deviation (SD) for continuous variables and frequencies with percentages for categorical variables. The normality of the Infertility Attitude Scale and Gender Perception Scale scores was assessed by examining skewness and kurtosis values. As both scale scores met the assumption of normality, parametric statistical tests were used throughout the analyses.

Differences in Infertility Attitude Scale and Gender Perception Scale scores according to sociodemographic and obstetric characteristics were analysed using the independent samples t test for two-group comparisons and one-way analysis of variance (ANOVA) for comparisons involving three or more groups. When ANOVA revealed statistically significant differences, pairwise comparisons were performed using the Least Significant Difference (LSD) post hoc test. The association between Gender Perception Scale and Infertility Attitude Scale scores was evaluated using Pearson’s correlation analysis. Simple linear regression analysis was performed to determine whether gender perception was a significant predictor of attitudes towards infertility, with the Infertility Attitude Scale score entered as the dependent variable and the Gender Perception Scale score as the independent variable. A two-sided p value < 0.05 was considered statistically significant.

## Results

A total of 346 women participated in the study. Table 1 presents the comparison of ATIS and GPS scores according to participants’ sociodemographic and pregnancy-related characteristics. ATIS scores differed significantly according to family type, educational level, place of residence, marital status, type of marriage, number of children, pregnancy history, and knowledge about infertility (all *p* < 0.05). Similarly, GPS scores differed significantly according to employment status, family type, educational level, place of residence, marital status, type of marriage, number of children, pregnancy history, knowledge about infertility, and pregnancy outcome (all *p* < 0.05) (Table 1).

**Table 1:** Comparison of Infertility Attitude Scale (ATIS) and Gender Perception Scale (GPS) scores according to women’s sociodemographic and pregnancy-related characteristics

Variable	Category	n	ATIS			GPS		
			Mean ± SD	Statistic (t/F)	p	Mean ± SD	Statistic (t/F)	p
Employment status	Unemployed	213	48.08 ± 7.66	-0.453	0.651	95.78 ± 18.99	-2.165	0.031*
	Employed	133	48.46 ± 7.41			100.12 ± 16.64		
Family type (*n* = 329)	Nuclear family	253	49.18 ± 7.08	4.938	< 0.001*	100.49 ± 16.82	6.358	< 0.001*
	Extended family	76	44.39 ± 8.42			86.04 ± 19.12		
Perceived income level	Income lower than expenses	98	47.86 ± 9.22	0.17	0.844	96.17 ± 19.19	1.352	0.260
	Income equal to expenses	185	48.34 ± 6.40			96.99 ± 18.24		
	Income higher than expenses	63	48.48 ± 7.90			100.78 ± 16.39		
Educational level	Literate ^A^	39	44.59 ± 5.76	11.911	< 0.001*	82.79 ± 16.74	22.657	< 0.001*
	Primary education ^B^	45	44.11 ± 9.09			86.27 ± 19.28		
	Secondary education ^C^	134	48.67 ± 7.41			100.11 ± 16.29		
	Bachelor’s degree or higher ^D^	128	50.31 ± 6.65			103.06 ± 16.18		
	Post hoc		C, D > A,B			C, D > A,B		
Place of residence	City ^A^	62	48.79 ± 6.34	10.74	< 0.001*	95.00 ± 14.50	26.58	< 0.001*
	District ^B^	223	49.15 ± 7.21			101.78 ± 16.48		
	Town/Village ^C^	61	44.28 ± 8.69			84.13 ± 20.82		
	Post hoc		A, B > C			B > A> C		
Marital status	Married ^A^	187	47.72 ± 7.75	17.771	< 0.001*	93.77 ± 18.62	28.346	< 0.001*
	Single ^B^	122	50.86 ± 6.18			107.04 ± 13.87		
	Other ^C^	37	42.97 ± 7.12			88.03 ± 15.79		
	Post hoc		B > A> C			B > A,C		
Type of marriage (*n* = 229)	Arranged marriage (without consent) ^A^	27	42.74 ± 8.45	8.67	< 0.001*	78.19 ± 19.36	22.532	< 0.001*
	Arranged marriage (with consent) ^B^	79	45.73 ± 8.13			88.49 ± 17.78		
	Marriage by mutual agreement ^C^	123	48.78 ± 7.07			99.50 ± 15.51		
	Post hoc		C > A,B			C > B> A		
Number of children (*n* = 182)	1 ^A^	38	50.05 ± 5.63	4.322	0.015*	100.87 ± 11.91	13.239	< 0.001*
	2 ^B^	68	49.18 ± 7.06			99.99 ± 16.44		
	3 or more ^C^	76	46.38 ± 7.94			86.88 ± 20.22		
	Post hoc		A, B > C			A, B > C		
Pregnancy history (*n* = 327)	No	121	49.55 ± 7.90	2.695	0.007*	104.66 ± 16.44	5.936	< 0.001*
	Yes	206	47.21 ± 7.34			92.78 ± 18.05		
Mode of conception (*n* = 205)	Spontaneous conception	170	47.52 ± 7.36	1.243	0.215	92.71 ± 18.70	-0.079	0.937
	Conception following infertility treatment	35	45.83 ± 7.28			92.97 ± 14.97		
Knowledge about infertility (*n* = 330)	No	102	47.77 ± 7.69	-3.602	< 0.001*	94.35 ± 19.41	-4.308	< 0.001*
	Yes	228	48.76 ± 7.44			101.04 ± 16.17		
Family history of infertility (*n* = 336)	No	208	49.30 ± 7.20	-0.112	0.911	100.71 ± 16.88	0.021	0.983
	Yes	128	48.12 ± 7.50			97.50 ± 19.02		
Pregnancy outcome (*n* = 204)	Miscarriage or stillbirth	44	45.50 ± 6.30	-1.832	0.068	87.68 ± 18.52	-2.074	0.039*
	Live birth	160	47.78 ± 7.54			94.01 ± 17.75		

*Values are presented as mean ± standard deviation (SD). Independent samples t-test or one-way analysis of variance (ANOVA) was used as appropriate. For statistically significant ANOVA results, post hoc comparisons were performed using the Least Significant Difference (LSD) test. *p < 0.05

A moderate positive correlation was found between ATIS and GPS scores (*r* = 0.634, *p* < 0.001) (Table 2).

**Table 2:** Correlation between attitudes towards infertility and gender perception

Variable	1	2
1.Infertility Attitude Scale (ATIS)	1	
2. Gender Perception Scale (GPS)	0.634**	1

** Pearson correlation coefficient; *p* < 0.001

The mean ATIS score was 48.23 ± 7.55, whereas the mean GPS score was 97.45 ± 18.22. Skewness and kurtosis values for both scales were within the acceptable range (± 2), supporting the assumption of normality (Table 3).

**Table 3:** Descriptive statistics for the infertility attitude scale and gender perception scale scores

Scale	Min	Max	Mean	SD	Skewness	Kurtosis
Infertility Attitude Scale (ATIS)	12	60	48.23	7.55	-0.976	1.463
Gender Perception Scale (GPS)	43	125	97.45	18.22	-0.683	-0.132

Multiple linear regression analysis showed that the overall model was statistically significant, F(15, 289) = 15.834, *p* < 0.001, explaining 46.8% of the variance in attitudes towards infertility (R² = 0.468; adjusted R² = 0.440). Gender perception was positively associated with attitudes towards infertility (B = 0.262, β = 0.621, 95% CI: 0.218–0.306, *p* < 0.001). Employed participants had lower attitude scores than unemployed participants (B = − 1.775, β = −0.111, *p* = 0.022), while divorced participants had lower scores than married participants (B = − 3.859, β = −0.114, *p* = 0.014). Participants with no pregnancy history also had lower attitude scores than those with a pregnancy history (B = − 2.599, β = −0.164, *p* = 0.030). Other variables were not significantly associated with infertility attitudes (*p* > 0.05) (Table 4).

**Table 4:** Multiple linear regression analysis of the association between gender perception, sociodemographic characteristics, and attitudes towards infertility

Predictor	B	SE	β	95% CI	t	*p*
GPS	**0.262**	**0.022**	**0.621**	**0.218–0.306**	**11.797**	**< 0.001**
Age	0.015	0.045	0.023	−0.074–0.104	0.341	0.734
Educational level						
Literate vs. Illiterate	−2.948	2.029	−0.096	−6.941–1.045	−1.453	0.147
Primary education vs. Illiterate	−3.606	1.830	−0.161	−7.209–−0.004	−1.970	0.050
Secondary education vs. Illiterate	−2.509	1.828	−0.158	−6.108–1.090	−1.372	0.171
Undergraduate/graduate vs. Illiterate	−0.910	1.863	−0.056	−4.577–2.757	−0.488	0.626
Employment status						
Employed vs. Unemployed	−1.775	0.771	−0.111	−3.293–−0.257	−2.302	0.022
Perceived income						
Income equal to expenses vs. lower than expenses	0.414	0.797	0.027	−1.155–1.982	0.519	0.604
Income higher than expenses vs. lower than expenses	0.170	1.040	0.009	−1.877–2.217	0.164	0.870
Place of residence						
City vs. District	1.032	0.897	0.051	−0.732–2.796	1.151	0.251
District vs. Province	−1.069	0.954	−0.066	−2.947–0.809	−1.120	0.263
Town/village vs. Province	−1.366	1.212	−0.069	−3.752–1.019	−1.127	0.261
Marital status						
Single vs. Married	0.649	1.355	0.038	−2.018–3.316	0.479	0.632
Cohabiting vs. Married	−2.437	1.709	−0.074	−5.800–0.926	−1.426	0.155
Divorced vs. Married	−3.859	1.563	−0.114	−6.934–−0.783	−2.470	0.014
Family type						
Extended vs. Nuclear family	−1.035	0.912	−0.057	−2.831–0.760	−1.135	0.257
Pregnancy history						
No vs. Yes	−2.599	1.189	−0.164	−4.938–−0.259	−2.186	0.030

Reference categories: unemployed, illiterate, income lower than expenses, province, married, nuclear family, and history of pregnancy

Model: F(15, 289) = 15.834, *p* < 0.001, R² = 0.468, adjusted R² = 0.440

*B* unstandardized regression coefficient, *SE* standard error, *β* standardized regression coefficient, *CI* confidence interval

## Di̇scussi̇on

This study examined the relationship between gender perception and attitudes towards infertility among women of reproductive age. The findings indicate that attitudes towards infertility were associated with gender perception as well as several sociodemographic characteristics. First, a significant positive correlation was identified between gender perception and attitudes towards infertility. Second, multiple linear regression analysis showed that gender perception remained positively associated with attitudes towards infertility after accounting for relevant sociodemographic and pregnancy-related characteristics, with the model explaining 46.8% of the variance in ATIS scores (R² = 0.468; adjusted R² = 0.440). Finally, several sociodemographic and obstetric characteristics were significantly associated with both attitudes towards infertility and gender perception. Collectively, these findings suggest that gender perception is an important factor associated with women’s attitudes towards infertility and underscore the importance of integrating gender-sensitive approaches into reproductive health services.

One of the main findings of this study was the identification of a moderate positive correlation between gender perception and attitudes towards infertility. Women with more egalitarian gender perceptions had more positive attitudes towards infertility. This finding is also supported by a recent study conducted among university students in a similar sociocultural context, in which more positive gender perceptions were significantly associated with more positive attitudes towards infertility [15]. This finding is consistent with previous studies reporting that individuals with more egalitarian gender role attitudes have greater fertility awareness, are less likely to endorse stigmatizing attitudes towards infertility, and exhibit more supportive attitudes towards individuals experiencing infertility [15, 16, 21]. Gender norms are considered one of the key factors influencing whether infertility is perceived, particularly among women, as a medical condition or as a sign of social inadequacy [18]. Likewise, social expectations surrounding motherhood and traditional gender roles have been reported to substantially influence women’s reproductive experiences and psychological well-being [6]. One possible explanation for the observed association is that gender perceptions may shape how women interpret reproductive roles, responsibility, and expectations surrounding childbearing. Traditional gender norms may position motherhood and fertility as central components of women’s identity, potentially increasing the tendency to interpret infertility through a gendered rather than exclusively biomedical framework. Recent qualitative evidence indicates that women may experience infertility-related stigma through prescribed gender roles, expectations of motherhood, and perceptions of responsibility or blame, illustrating the close relationship between gender norms and infertility-related stigma (Bornstein et al. [22]). These findings suggest that women with more egalitarian gender perceptions tend to have more positive attitudes towards infertility, which may reflect greater resistance to infertility-related stigmatizing beliefs.

The multiple linear regression analysis further demonstrated a significant positive association between gender perception and attitudes towards infertility after accounting for relevant sociodemographic and pregnancy-related characteristics. Gender perception remained strongly associated with infertility attitudes (B = 0.262, β = 0.621, 95% CI: 0.218–0.306, *p* < 0.001), and the overall model explained 46.8% of the variance in ATIS scores (R² = 0.468; adjusted R² = 0.440). However, these findings should be interpreted as observed statistical associations rather than evidence of causal relationships. Because of the cross-sectional design, the temporal sequence between gender perception and attitudes towards infertility could not be established, and it is therefore not possible to determine whether gender perceptions precede or develop concurrently with attitudes towards infertility. The proportion of explained variance indicates that gender perception represents an important psychosocial factor associated with infertility attitudes. Although studies directly examining the association between gender perception and attitudes towards infertility are limited, previous research has shown that traditional gender norms negatively affect the quality of life, psychological well-being, and infertility experiences of individuals experiencing infertility [3, 18]. In addition, gender norms have been identified as important social determinants influencing women’s health behaviours, healthcare utilisation, and reproductive health decision-making [5]. A systematic review demonstrated that gender norms can shape women’s sexual and reproductive health by influencing autonomy, access to reproductive health information and services, and responses to culturally embedded social expectations and stigma. The review also emphasized that these effects vary across sociocultural contexts, highlighting the importance of culturally responsive approaches to reproductive healthcare [13]. The observed association may therefore reflect the interaction of individual beliefs with broader social and cultural expectations rather than a direct effect of gender perception on infertility attitudes. Gender norms are embedded within family relationships, educational experiences, economic participation, and healthcare environments, and their influence may differ across sociocultural settings [13]. These findings highlight the potential value of educational and counselling approaches that address gender norms and infertility-related stigma within reproductive healthcare. However, longitudinal and intervention studies are needed to determine whether changes in gender-related beliefs are accompanied by changes in infertility attitudes.

In the present study, the mean ATIS score was 48.23 ± 7.55, indicating that the participants generally held positive attitudes towards infertility. Consistent with the present findings, studies conducted among university students and women of reproductive age have reported moderate to high levels of positive attitudes towards infertility [16, 23, 24]. This may reflect the increasing recognition of infertility as an important component of reproductive health, together with increasing awareness of infertility-related issues. Furthermore, previous research has shown that greater knowledge about infertility contributes to correcting misconceptions and reducing stigmatizing attitudes [14]. In this context, improving access to accurate information about infertility and expanding educational interventions may help foster more positive attitudes towards infertility.

In the present study, the mean GPS score was 97.45 ± 18.22, indicating that the participants generally had egalitarian gender perceptions. Although studies simultaneously examining gender perception and attitudes towards infertility are limited, previous research has shown that gender perception is closely associated with women’s educational attainment, participation in the workforce, empowerment, and reproductive health experiences [12, 13]. In addition, more egalitarian gender perceptions have been associated with improved access to reproductive health services and more active participation in health-related decision-making among women [5]. Taken together, these findings suggest that policies promoting gender equality may not only contribute to women’s empowerment but also foster more positive attitudes towards infertility.

In the present study, both attitudes towards infertility and gender perception differed significantly according to several sociodemographic characteristics. In particular, educational level, employment status, family type, place of residence, marital status, and type of marriage were significantly associated with both attitudes towards infertility and gender perception. These univariate differences highlight the relevance of sociodemographic context when interpreting women’s attitudes towards infertility and gender perceptions. However, the multiple regression analysis showed that not all of these characteristics remained significantly associated with infertility attitudes after their simultaneous inclusion in the model. This finding reinforces the view that infertility is not merely a biomedical condition but also a multidimensional phenomenon shaped by the social and cultural context in which individuals live [8]. Higher educational attainment was associated with more positive attitudes towards infertility and more egalitarian gender perceptions. This association may be explained by the role of education in enabling individuals to critically examine gender norms, improve health literacy, and access evidence-based reproductive health information. Consistent with this finding, studies examining the relationship between gender norms and women’s access to reproductive health services have reported that higher educational attainment is associated with more egalitarian gender perceptions and more favourable reproductive health outcomes [12, 13]. Likewise, Mabitsela et al. (2025) reported that educational attainment was associated with greater infertility-related knowledge and awareness [14]. The findings related to employment status further support the influence of women’s economic and social empowerment on gender perception. In the present study, employed women had significantly higher gender perception scores than unemployed women. Previous research has shown that economic independence increases women’s participation in decision-making and facilitates questioning of traditional gender norms [25]. Similarly, women’s economic empowerment has been associated not only with improved individual well-being but also with greater awareness of gender equality and increased participation in social innovation processes [26]. Therefore, promoting women’s access to education and employment may represent an important component of policies aimed at advancing gender equality.

The findings related to place of residence and family structure further highlight the influence of the social environment on women’s attitudes and gender perceptions. The higher gender perception scores observed among women living in urban areas may be associated with greater access to educational opportunities, healthcare services, and reliable sources of information accompanying urbanisation. The importance of regional context in reproductive health has also been demonstrated in assisted reproduction research. Rodrigues-Martins et al. [27] reported differences in assisted reproduction treatment outcomes according to women’s region of residence, even within the same country and at the same tertiary fertility center, highlighting the potential contribution of regional sociodemographic and socioeconomic factors to reproductive health experiences [26]. Similarly, studies conducted in Türkiye have reported that women residing in urban areas tend to have more egalitarian gender perceptions [28]. In contrast, the stronger persistence of traditional gender norms in rural settings may influence both women’s gender perceptions and their attitudes towards infertility [29]. International evidence further illustrates why demographic characteristics and contextual factors should be considered when interpreting reproductive experiences. In a five-year cohort study of 5,250 infertile couples receiving their first in vitro fertilization treatment at public fertility centers in Northern and Central Portugal, female age was identified as an important factor associated with treatment outcomes [30]. Although this study focused on assisted reproductive treatment outcomes rather than gender perceptions or attitudes towards infertility, it highlights the relevance of demographic characteristics within infertility care and demonstrates how evidence from different populations can broaden the interpretation of reproductive experiences [31]. Taken together, these findings suggest that fostering positive attitudes towards infertility requires not only individual educational initiatives but also broader social policies that strengthen women’s access to education, employment, and healthcare services.

In the present study, type of marriage, number of children, pregnancy history, and knowledge about infertility were significantly associated with both attitudes towards infertility and gender perception. Notably, women who had married by mutual agreement had higher scores for both attitudes towards infertility and gender perception than those who had entered arranged marriages. This finding may be explained by stronger communication, mutual support, and shared decision-making in marriages established through individual choice. Indeed, compared with arranged marriages, marriages based on mutual acquaintance and agreement have been associated with greater emotional closeness and trust between spouses [31]. This more egalitarian relationship dynamic may, in turn, facilitate the development of more egalitarian gender perceptions among women and contribute to more positive attitudes towards infertility.

In the present study, women with a greater number of children had lower scores for both attitudes towards infertility and gender perception. Preferences regarding childbearing are shaped not only by individual choices but also by cultural values, family structures, and societal expectations [32]. Likewise, previous research has reported that more traditional gender role attitudes are associated with a greater desire to have children and a stronger preference for male offspring [33]. These findings may indicate that, in societies where motherhood is regarded as a central component of women’s identity, larger family size may be associated with more traditional gender-role orientations.

In the present study, having a history of pregnancy was associated with higher scores for both attitudes towards infertility and gender perception. Improved access to reproductive and maternal health services has been reported to support informed reproductive decision-making and better reproductive health outcomes among women [34]. These findings suggest that comprehensive reproductive health counselling provided throughout the life course may not only improve reproductive health outcomes but also contribute to more egalitarian gender perceptions and more positive attitudes towards infertility.

Women who were knowledgeable about infertility had higher gender perception scores and more positive attitudes towards infertility, suggesting that knowledge may play an important role in reducing stigmatizing beliefs. Similarly, a study conducted among women with infertility reported that higher gender perception was associated with lower levels of infertility-related psychological distress [17]. These findings suggest that access to accurate information about infertility may not only improve women’s understanding of infertility but also help them view infertility from a more realistic and less stigmatizing perspective. Taken together, the findings contribute to the existing literature by providing community-based evidence on the association between gender perception and attitudes towards infertility among women of reproductive age. This study has several strengths. First, it examined the relationship between gender perception and attitudes towards infertility using established measurement instruments in a sample of 346 women of reproductive age. Second, the study considered a range of sociodemographic and reproductive characteristics, allowing the findings to be interpreted within a broader social and reproductive health context. Third, the use of correlation and regression analyses provided a quantitative assessment of the strength of the association between gender perception and attitudes towards infertility. Finally, conducting the study within a primary healthcare setting enhances its practical relevance for reproductive health counselling.

In the present study, women whose pregnancies had resulted in a live birth had higher gender perception scores. Although studies directly examining this association are limited, previous research has demonstrated that gender norms influence women’s experiences of motherhood, reproductive health, and perceptions of women’s identity [3, 6, 18]. These findings suggest that women’s reproductive experiences may be shaped not only by biological processes but also by gender norms and the cultural meanings attached to motherhood. Therefore, healthcare professionals working in the field of infertility should not limit their role to the provision of clinical care but should also integrate gender-sensitive approaches into reproductive healthcare services. Finally, these findings should be interpreted within the sociocultural context of Türkiye. Gender norms, family expectations, marriage practices, educational opportunities, and access to reproductive health information may differ substantially across societies and may influence how infertility is understood and evaluated. A systematic review has demonstrated that the effects of gender norms on women’s reproductive health vary across sociocultural contexts, supporting the need for culturally responsive approaches to reproductive healthcare [13]. Therefore, the present findings should not be assumed to be directly generalizable to women in other countries or cultural settings. Future multicentre and cross-cultural studies should examine whether the association between gender perception and attitudes towards infertility is consistent across different sociocultural environments and should investigate potential pathways involving infertility stigma, reproductive autonomy, social support, and gender-role expectations.

### Limitation of study

This study has several limitations. First, because participants were recruited from a single district in Çankırı Province using non-probability convenience sampling, the findings may not be generalizable to women in Çankırı Province or Türkiye as a whole. Second, the cross-sectional design precludes any causal inference regarding the relationship between gender perception and attitudes towards infertility. In particular, the cross-sectional design does not allow the temporal sequence between gender perception and attitudes towards infertility to be established. Thus, the direction of the observed association remains uncertain, and longitudinal studies are needed to clarify temporality and determine whether changes in gender perceptions are associated with subsequent changes in attitudes towards infertility. Third, because the data were collected using self-reported questionnaires, social desirability bias cannot be excluded. Participants may have been inclined to report more socially acceptable or egalitarian gender perceptions and more positive attitudes towards infertility, particularly given the socially sensitive nature of gender roles and infertility. Such reporting tendencies may have resulted in an overestimation or underestimation of the observed associations. Although the questionnaires were administered under conditions intended to protect confidentiality, the possibility of social desirability bias cannot be completely ruled out. Finally, the inclusion of women only limited the evaluation of gender perceptions related to infertility at the couple level. Future longitudinal studies involving both women and men from diverse geographical and sociocultural settings are needed to provide a more comprehensive understanding of the relationship between gender perception and attitudes towards infertility.

## Conclusions

This study demonstrated a significant positive association between gender perception and attitudes towards infertility among women and identified gender perception as a significant predictor of attitudes towards infertility. In addition, several sociodemographic and obstetric characteristics were significantly associated with both gender perception and attitudes towards infertility. These findings suggest that attitudes towards infertility are shaped not only by individual characteristics but also by gender norms and the broader sociocultural context in which women live.

Integrating gender-sensitive approaches into infertility counselling and reproductive healthcare services may contribute to the development of more positive and supportive attitudes towards infertility among women. In the Turkish context, reproductive health programs could incorporate gender-sensitive counselling that addresses gender-role expectations, infertility-related stigma, and perceptions of responsibility for infertility. Training midwives, nurses, and other reproductive health professionals in gender-sensitive and nonjudgmental communication may support more equitable and woman-centred care. Involving male partners more actively in infertility education and counselling may also help reduce the perception that infertility is primarily a woman’s responsibility. In societies where traditional gender norms remain influential, expanding educational and counselling programmes that promote reproductive rights, gender equality, and infertility awareness may help reduce stigma and improve women’s reproductive health experiences. These findings also have important implications for women’s health nursing and midwifery practice. Integrating the assessment of gender perception into reproductive healthcare may facilitate gender-sensitive counselling, promote woman-centred care, and improve women’s reproductive well-being.

Future longitudinal and qualitative studies involving populations from diverse sociocultural settings are warranted to further elucidate the relationship between gender perception and attitudes towards infertility.

## Supplementary Information

Supplementary Material 1.

## Data Availability

The datasets used and/or analysed during the current study are available from the corresponding author on reasonable request.

## Abbreviations

ATIS: Attitudes Toward Infertility Scale
GPS: Gender Perception Scale
SD: Standard Deviation
SE: Standard Error
ANOVA: Analysis of Variance
LSD: Least Significant Difference
SPSS: Statistical Package for the Social Sciences
