# Supplementary material for: Gender perception as a predictor of women’s attitudes towards infertility: a cross-sectional study
Source: BMC Womens Health. 2026 Sep 21;26:489. doi: 10.1186/s12905-026-04906-0 (PMC13628946; doi:10.1186/s12905-026-04906-0)
Supplement: Supplementary file 1 [file 12905_2026_4906_MOESM1_ESM.pdf]

Introductory Information Form

A. Sociodemographic Characteristics

1. What is your educational level?

1. No formal education
2. Literate
3. Primary education
4. Secondary education
5. Bachelor's degree or higher

2. What is your employment status?

1. Unemployed
2. Employed

3. *How would you describe your income status?*

1. Income is lower than expenses
2. Income is equal to expenses
3. Income is higher than expenses

4. Where do you live?

1. City
2. District
3. Town/village

5. What is your marital status?

1. Married
2. Single
3. Other: ________________________

6. *How did you get married?*

1. Arranged marriage without my consent
2. Arranged marriage with my consent
3. Marriage by mutual acquaintance and agreement

7. *What is your family type?*

1. Nuclear family
2. Extended family

B. Obstetric Characteristics

8. *Have you ever been pregnant?*
1. Yes
2. No

9. *How did you become pregnant?*
1. Spontaneously
2. Conception following infertility treatment

10. *What was the outcome of your previous pregnancy?*
1. Miscarriage or stillbirth
2. Live birth

11. How many children do you have?
1. 1
2. 2
3. 3 or more

C. Questions Related to Infertility

12. Do you have any knowledge about infertility (being unable to have children)?
1. Yes
2. No

13. Do you have any infertile individuals among your family members or close relatives?
1. Yes
2. No
